# Supplementary material for: Fine Mapping and Identification of a Candidate Gene for the Glossy Green Trait in Cabbage (Brassica oleracea var. capitata)
Source: Plants (Basel). 2023 Sep 21;12(18):3340. doi: 10.3390/plants12183340 (PMC10538046; doi:10.3390/plants12183340)
Supplement: Supplementary file 1 [file plants-12-03340-s001.zip › Figure S1.pdf]

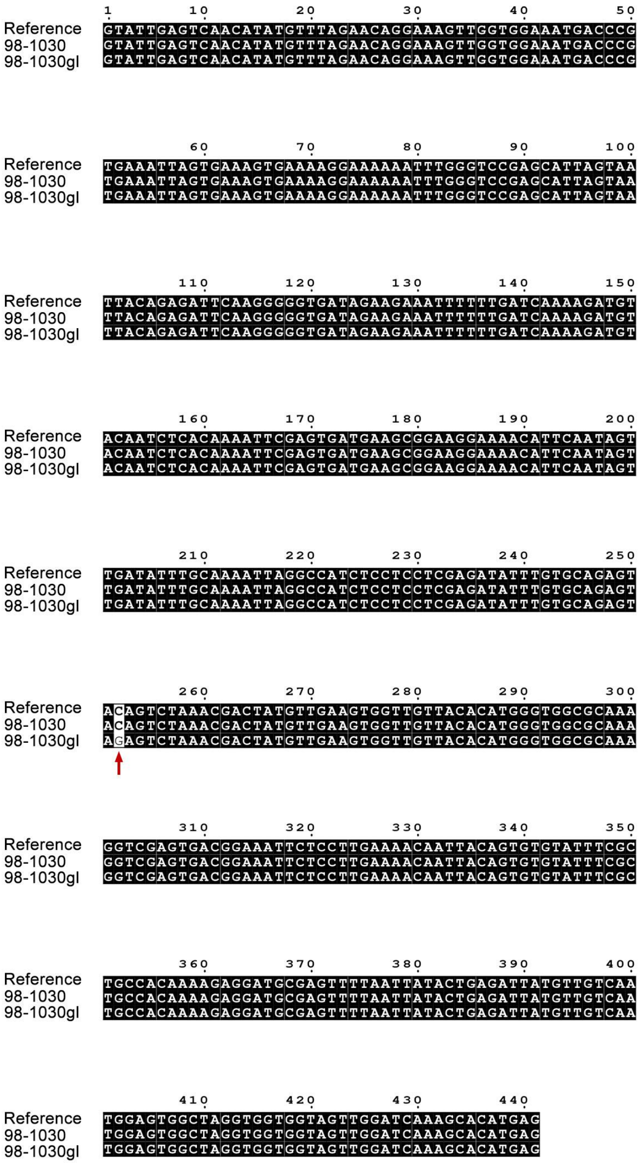

Figure S1. Sequence alignment of the second exon region of *Bol026949* in 98-1030 and 98-1030gl.

The red arrow indicates the SNP mutation (C→G) identified from BSA-seq analysis.
